# Isolation and characterization of non-O157 Shiga toxin-producing *Escherichia coli* from beef carcasses, cuts and trimmings of abattoirs in Argentina

**DOI:** 10.1371/journal.pone.0183248

**Published:** 2017-08-22

**Authors:** Victoria Brusa, Viviana Restovich, Lucía Galli, David Teitelbaum, Marcelo Signorini, Hebe Brasesco, Alejandra Londero, Diego García, Nora Lía Padola, Valeria Superno, Marcelo Sanz, Sandra Petroli, Magdalena Costa, Mariana Bruzzone, Adriana Sucari, Marcela Ferreghini, Luciano Linares, Germán Suberbie, Ricardo Rodríguez, Gerardo A. Leotta

**Affiliations:** 1 IGEVET—Instituto de Genética Veterinaria “Ing. Fernando N. Dulout” (UNLP-CONICET LA PLATA), Facultad de Ciencias Veterinarias UNLP, La Plata, Argentina; 2 Laboratorio de Microbiología de Alimentos, Facultad de Ciencias Veterinarias UNLP, La Plata, Argentina; 3 IPCVA–Instituto de Promoción de la Carne Vacuna Argentina, Ciudad Autónoma de Buenos Aires, Argentina; 4 CONICET—EEA Rafaela, Instituto Nacional de Tecnología Agropecuaria (INTA), Santa Fe, Argentina; 5 CIVETAN–Centro de Investigación Veterinaria Tandil (CONICET, CICPBA), Facultad de Ciencias Veterinarias, UNCPBA, Tandil, Argentina; 6 Centro Estudios Infectológicos “Dr. Daniel Stamboulian”, División Alimentos, Ciudad Autónoma de Buenos Aires, Argentina; 7 SENASA–Servicio Nacional de Sanidad y Calidad Agroalimentaria, Ciudad Autónoma de Buenos Aires, Argentina; 8 Instituto de Economía (CICPES, INTA), Ciudad Autónoma de Buenos Aires, Argentina; Cornell University, UNITED STATES

## Abstract

Several foods contaminated with Shiga toxin-producing *Escherichia coli* (STEC) are associated with human diseases. Some countries have established microbiological criteria for non-O157 STEC, thus, the absence of serogroups O26, O45, O103, O104, O111, O121, and O145 in sprouts from the European Union or ground beef and beef trimmings from the United States is mandatory. While in Argentina screening for O26, O103, O111, O145 and O121 in ground beef, ready-to-eat food, sausages and vegetables is mandatory, other countries have zero-tolerance for all STEC in chilled beef. The aim of this study was to provide data on the prevalence of non-O157 STEC isolated from beef processed in eight Argentinean cattle slaughterhouses producing beef for export and local markets, and to know the non-O157 STEC profiles through strain characterization and genotypic analysis. Samples (n = 15,965) from 3,205 beef carcasses, 9,570 cuts and 3,190 trimmings collected between March and September 2014 were processed in pools of five samples each. Pools of samples (n = 3,193) from 641 carcasses, 1,914 cuts and 638 trimming were analyzed for non-O157 STEC isolation according to ISO/CEN 13136:2012. Of these, 37 pools of carcasses (5.8%), 111 pools of cuts (5.8%) and 45 pools of trimmings (7.0%) were positive for non-O157 STEC. STEC strains (n = 200) were isolated from 193 pools of samples. The most prevalent serotypes were O174:H21, O185:H7, O8:H19, O178:H19 and O130:H11, and the most prevalent genotypes were *stx*_2c(vh-b)_ and *stx*_2a_/*saa*/*ehxA*. O103:H21 strain was *eae*-positive and one O178:H19 strain was *aggR*/*aaiC*-positive. The prevalence of non-O157 STEC in beef carcasses reported here was low. None of the non-O157 STEC strains isolated corresponded to the non-O157 STEC serotypes and virulence profiles isolated from human cases in Argentina in the same study period. The application of microbiological criteria for each foodstuff should be determined by risk analysis in order to have a stringent monitoring system. Likewise, zero-tolerance intervention measures should be applied in beef, together with GMP and HACCP. Further, collaborative efforts for risk assessment, management and communication are extremely important to improve the safety of foodstuffs.

## Introduction

Foodborne diseases are caused by ingestion of foodstuffs contaminated with microorganisms or chemicals, and are considered a growing and global public health problem [1]. Since ruminants are a reservoir of Shiga toxin-producing *Escherichia coli* (STEC), contaminated foodstuffs derived from cattle have been responsible for human illness worldwide [2–4]. However, other STEC-contaminated foods such as leafy vegetables, dairy products, fruits and other meat shave also been associated with human diseases [5, 6]. The combinations of markers required by STEC to cause clinical infections are not clear, however strains harboring *stx*_2_/*eae* are associated with higher risk for more serious illness [7]. Although 1,152 different serotypes have been described since the first published report of STEC serotypes in 1980 [8], it is not possible to predict their potential to cause disease [7].

STEC is the primary etiological agent of post-enteric hemolytic uremic syndrome (HUS), which is endemic in Argentina. During 2015, 337 HUS cases were reported [9], and even though *E*. *coli* O157:H7 was the predominant serotype isolated from patients, non-O157 STEC strains were responsible for 25.1% of STEC infections [9]. The main non-O157 STEC serotypes and virulence profiles isolated from ill patients were O145:NM *stx*_2a_/*eae*/*ehxA* and O121:H19 *stx*_2a_/*eae*/*ehxA*.

Some countries have established microbiological criteria for non-O157 STEC detection. In the United States (US), the absence of detectable O26, O45, O103, O111, O121 and O145 serogroups in ground beef and beef trimmings is mandatory [10]. In the European Union (EU), sprouts are analyzed for the absence of O26, O103, O104, O111 and O145 STEC serogroups[11]. Recently, the Argentinean Food Code (AFC) included the screening for O26, O103, O111, O145 and O121 serogroups in ground beef, ready-to-eat food, sausages and vegetables[12]. Other countries have implemented the zero tolerance policy for all STEC in chilled beef[13].

Considering the clinical relevance and risk of *E*. *coli* O157:H7 in food [7], its absence in beef is mandatory. However, knowledge about a defined combination of virulence factors required for clinical infections associated with all non-O157 STEC serotypes is not enough. The epidemiological relationship of beef contaminated with any non-O157 STEC serotype and human disease is still difficult to assess. Thus, further studies about the genotypic profile of non-O157 STEC strains present in beef could contribute to determining more accurately the importance of meat as a bacterial source of human STEC infection and could help avoid unnecessary trade disputes.

The aim of this study was to provide data on the prevalence of non-O157 STEC isolated from beef processed in Argentinean exporting abattoirs under current commercial operation practices, and to know the non-O157 STEC virulence profiles through strain characterization and genotypic analysis.

## Materials and methods

### Abattoir selection and sample collection

Samples (n = 15,965) from beef carcasses (n = 3,205), cuts (n = 9,570) and trimmings (n = 3,190) were collected at cattle slaughterhouses producing beef for export and local markets of Argentina between March and September 2014. Eight abattoirs were invited to participate voluntarily in this study and were identified as A to H. They were selected considering the number of cattle slaughtered over a five-year period (more than 800,000) and their geographic location (Buenos Aires, 33°46′S 60°05′W; 34°18′S 60°15′W; 34°25′S 58°35′W; 34°53′S 58°02′W; Santa Fé, 32°57′S 60°39′W; 29°14′S 59°56′W; 33°48′S 61°20′W; San Luis, 33°40′S 65°28′W). Sampling was approved by the National Service of Agrifood Health and Quality of Argentina (SENASA, for its Spanish acronym). All samples presented the organoleptic and commercial characteristics established in National Decree No 4238/68 for meats[14].

Sample size was calculated taking into account an estimated 9.0% prevalence of non-O157 STEC in Argentinean beef carcasses (97.5% confidence level and <1% precision) [15].

Before sampling, each abattoir participated in a training program to ensure the systematic collection and processing of samples. This program included the person responsible for quality control, all samplers and the SENASA official veterinarian in each abattoir. Details of sample collection are shown in Table 1.

**Table 1:** Pools, total number and type of beef samples analyzed in the eight abattoirs.

	Half carcass		Loin		Striploin		Heart of rump		Trimmings		Total	
Abattoir	Pools	n	Pools	n	Pools	n	Pools	n	Pools	n	Pool	n
A	74	370	76	380	76	380	76	380	76	380	378	1,890
B	74	370	73	365	73	365	73	365	73	365	366	1,830
C	71	355	70	350	70	350	70	350	70	350	351	1,755
D	74	370	74	370	74	370	74	370	74	370	370	1,850
E	130	650	128	640	128	640	128	640	128	640	642	3,210
F	74	370	74	370	74	370	74	370	74	370	370	1,850
G	81	405	81	405	81	405	81	405	81	405	405	2,025
H	63	315	62	310	62	310	62	310	62	310	311	1,555
Total samples	641	3,205	638	3,190	638	3,190	638	3,190	638	3,190	3,193	15,965

Cattle came from 357 cities of 14 Argentinean provinces. The sampled half carcasses were from cattle typified according to the grading system of SENASA as steers (64.3%), cows (26.4%), young steers (7.7%), heifers (0.9%) and calves (0.7%). Animals came from extensive breeding systems (72.6%), intensive breeding systems (19.8%) and fairs (7.6%).

Samples of carcasses (n = 5), loin (n = 5), striploin (n = 5), heart of rump (n = 5) and trimmings (n = 5) were collected daily from March to September, on the first and third week of each month in the eight abattoirs.

Beef carcass samples were obtained prior to entering the chilling rooms. The carcass surface (covering a total half carcass, including the anterior region and the posterior lateral hock, round, and rump of the posterior region) was swabbed with a sterile sponge (Whirl-Pak speci-sponge, Nasco, USA) previously soaked in 10 ml buffered peptone water (Biokar, Zac de Ther, France). The posterior area was first swabbed with ten strokes of the sponge in two directions. The sponge was then rotated and the anterior area was covered by another ten strokes in both directions. After swabbing, sponges were placed into sterile stomacher bags and stored at 4°C until processing.

Beef samples (loin, striploin and heart of rump) were obtained after carcass deboning with a sterile sponge (Whirl-Pak) soaked in 10 ml buffered peptone water (Biokar) every 2 h. The total surface of each cut was sampled by swabbing with ten strokes of the sponge in two directions. After swabbing, sponges were returned into sterile stomacher bags and stored at 4°C until processing. Five sponges from each cut were collected after the 8-h sampling period.

Trimming samples (n = 5, 200 g each) from bags located in deboned carcasses were collected every 2 h. After this 8-h sampling period, samples were pooled (1,000 g in total) and stored at 4°C.

This sampling scheme was chosen in order to increase the sensitivity by swabbing a total surface of carcasses and cuts and by washing trimmings, thus encompassing 100% of surfaces from each sample.

### Bacteriological analysis

A total of 3,193 pools of samples from 641 carcasses, 1,914 cuts and 638 trimmings were processed in pools of five samples each (Table 1).

All samples were analyzed for non-O157 STEC according to ISO/CEN 13136:2012 [16], with some modifications. Briefly, each sampling day five sponges from carcass samples were put into a stomacher bag and 500 ml of modified trypticase soy broth containing 8 mg/L novobiocin plus casamino acids (mTSB-8, AcumediaManufacturers, Lansing, MI) was added. Each sponge was mixed in the stomacher bag for 2 min and incubated for 20 h at 41.5°C. The same procedure was followed with sponges from cut pools. In addition, 400 ml mTSB-8 was added to each sample containing 1000 g of trimmings and further washed by inverting vigorously the bag. The wash material was disposed into sterile stomacher bags and incubated for 20 h at 41.5°C.

One ml of each sample was plated onto three Mac Conkey agar (Becton Dickinson Co., Sparks, MD, USA) and three Levine-eosin methylene blue agar (Biokar) plates. In order to obtain well-isolated discrete colonies, 1 ml of the enriched sample was diluted streaking it over successive quadrants of three plates of each culture media. All plates were incubated for 18 h at 37°C. Fifty colonies with *E*. *coli* morphology were selected and point-inoculated in nutrient agar (NA, Laboratorios Britania, Buenos Aires, Argentina). After incubation, five pools of 10 colonies each were screened for *stx*_1_ and *stx*_2_ genes by multiplex-PCR [17]. Colonies from positive pools were analyzed individually by multiplex-PCR for the detection of the *stx*-positive colony. Presumptive non-O157 STEC colonies were isolated in NA (Laboratorios Britania), confirmed by multiplex PCR and stored in nutrient broth (NB, Laboratorios Britania) with 40% glycerol at -70°C for further characterization.

### Biochemical tests, serotyping and genotypic characterization of isolates

The isolated strains were characterized by biochemical tests according to Ewing [18]. STEC serotyping of O and H antigens was performed as described by Blanco et al. [19]. For genotypic characterization, the following virulence and adherence genes were tested by PCR: *eae* (intimin) [20], *ehxA* (enterohemolysin) [21], *saa* (Shiga toxin-producing *E*. *coli* autoagglutinating adhesion), *efa* (enterohemorrhagic *E*. *coli* factor for adherence), *toxB* (protein involved in adherence), *iha* (iron-regulated gene A homolog adhesion similar to *V*. *cholerae*) [22], *subAB* (subtilase cytotoxin) [23], *cdt-V* (cytolethal distending toxin) [24] and *astA* (enteroaggregative *E*. *coli* heat-stable toxin) [25]. Also, *aggR* (transcriptional activator of aggregative adherence fimbria I expression of enteroaggregative *E*. *coli*) and *aaiC* (protein secreted by enteroaggregative *E*. *coli*) genes were detected by real-time PCR[26].

### Molecular subtyping of strains

STEC subtypes *stx*_1_ and *stx*_2_ were analyzed by PCR [27]. DNA fragments obtained by PCR [28] were analyzed by restriction fragment length polymorphism (RFLP).

Subtyping of *E*. *coli* non-O157 was performed by pulsed-field gel electrophoresis (PFGE) using the one day (24–26 h) PulseNet standardized laboratory protocol [29].

Restriction digestion of DNA in agarose plugs was carried out with *Xba*I and *Xma*JI (*Bln*I) as primary and secondary enzymes, respectively (Thermo Scientific, MA, USA). PFGE images of gels were obtained by MaestroGen slider imager (Maestrogen Inc., Nevada, USA). Tagged image file format (TIFF) images were analyzed with BioNumerics, version 6.6 software package (Applied Maths, Sint-Martens-Latem, Belgium) using the Dice coefficient and the unweighted pair group method with arithmetic mean (UPGMA) to generate dendrograms with 1.5% band matching tolerance. Two or more isolates with identical *Xba*I-PFGE pattern (100% similarity) were grouped in a cluster.

### Statistical analyses

Multivariable logistic regression analyses were performed using a generalized linear mixed model (GLMM) and abattoir as random effect. It was evaluated the effect of each cut (loin, striploin, heart of rump or trimmings) on the binary outcome variable (positive/negative) of non-O157 STEC. Also, were evaluated the effect of the grading of animals (adult animals, steers + cows; young animals, young steers, heifers and calves) and production system (intensive breeding, extensive breeding and fairs) on the binary outcome variable (positive/negative) of non-O157 STEC in carcasses. All statistical analyses were performed with InfoStat software (Universidad Nacional de Córdoba) using a significance *p*<0.05.

## Results

From the 3,193 pools of samples analyzed in the eight abattoirs, 193 (6.0%) were positive for non-O157 STEC, as follows: carcass, 37 (5.8%) pools of samples; cuts, 111 (5.8%) pools of samples; trimmings, 45 (7.0%) pools of samples. A total of 200 non-O157 STEC were isolated and characterized by biochemical test, serotype, genotype and *Xba*I-PFGE analysis. Their presence in each abattoir varied from 1.0 to 13.5% (Table 2) and more than one strain was isolated in seven out of the 193 (3.6%) pools of samples (Table 3).

**Table 2:** Number of pools of beef samples analyzed in the eight abattoirs and percentage of pools positive for non-O157 STEC.

Abattoirs	Pools of samples			Isolates
	Total	Positive	%	
**A**	378	15	4.0	15
**B**	366	15	4.1	15
**C**	351	19	5.4	21
**D**	370	50	13.5	54
**E**	642	26	4.0	26
**F**	370	28	7.6	28
**G**	405	38	9.1	39
**H**	311	2	1.0	2
**TOTAL**	3,193	193	6.0	200

**Table 3:** Prevalence of non-O157 STEC strains in beef carcass, loin, striploin, heart of rumpand trimmings in the eight abattoirs.

Sample category	Number of pools of samples	Number of positive pools	Positive pools (%)	95% CI^a^	Number of strains isolated	Number of pools with more than one serotype/total pool of samples	Total pool of samples (%)
Carcass	641	37	5.8	4.2–7.9	41	8/37	21.6
Loin	638	29	4.5	3.2–6.5	31	2/29	6.9
Striploin	638	37	5.8	4.2–7.9	37	0/37	0.0
Heart of rump	638	45	7.1	5.3–9.5	46	1/45	2.2
Trimmings	638	45	7.1	5.3–9.5	45	0/45	0.0
TOTAL	3193	193	6.0	5.3–6.9	200	11/193	5.7

^a^ 95%CI, 95% confidence interval

Comparison of the prevalence of non-O157 STEC in the different beef cuts and trimmings did not show significant differences (*P* = 0.185) (Table 3). Likewise, no differences were observed in the prevalence of non-O157 STEC in beef carcasses considering animal classification (*P* = 0.527) and type of farming (*P* = 0.264).

However, the number of non-O157 STEC-positive carcasses, loin, striploin, heart of rump and trimmings as well as the prevalence of non-O157 STEC differed among abattoirs (carcasses, *P*<0.001; loin, *P* = 0.017; striploin, *P*<0.001; heart of rump, *P* = 0.013; trimmings, *P* = 0.053) (Table 4).

**Table 4:** Number and percentage of beef carcass, loin, striploin, heart of rump and trimmings positive for non-O157 STEC according to abattoir.

	Carcass		Loin		Striploin		Heart of rump		Trimmings	
Abattoirs	Positive samples/total	%	Positive samples/total	%	Positive samples/total	%	Positive samples/total	%	Positive samples/total	%
**A**	2/74	2.7	2/76	2.6	2/76	2.6	5/76	6.6	4/76	5.3
**B**	0/74	0.0	2/73	2.7	3/73	4.1	3/73	4.1	7/73	9.6
**C**	9/71	12.7	2/70	2.9	4/70	5.7	2/70	2.9	3/70	4.3
**D**	14/74	18.9	6/74	8.1	12/74	16.2	9/74	12.2	9/74	12.2
**E**	4/130	3.1	3/128	2.3	3/128	2.3	7/128	5.5	9/128	7.0
**F**	7/74	9.5	5/74	6.8	3/74	4.1	10/74	13.5	3/74	4.1
**G**	0/81	0.0	9/81	11.1	10/81	12.3	9/81	11.1	9/81	11.1
**H**	1/63	1.6	0/62	0.0	0/62	0.0	0/62	0.0	1/62	1.6
**TOTAL**	**37/641**	**5.8**	**29/638**	**4.5**	**37/638**	**5.8**	**45/638**	**7.1**	**45/638**	**7.1**

### Biochemical test and serotyping

Among the 200 non-O157 STEC isolates, 175 belonged to 31 O-groups (O8, O15, O20, O22, O39, O41, O46, O48, O60, O73, O74, O82, O83, O84, O88, O91, O103, O113, O130, O141, O149, O154, O163, O164, O171, O174, O178, O179, O181, O183, O185), and 25 isolates were non-typeable (ONT). Nineteen H antigens (H2, H4, H7, H8, H11, H16, H18, H19, H21, H25, H26, H27, H28, H38, H41, H42, H46, H49, H66) were determined in 196 strains, three strains were non motile (NM) and one was non-typeable (HNT). Non-O157 STEC strains were grouped into 44 different serotypes, five of which were the most prevalent: O174:H21 (n = 22), O185:H7 (n = 19), O8:H19 (n = 17), O178:H19 (n = 15) and O130:H11 (n = 12). They represented 42.5% of the isolates and were isolated from all types of samples. Eighteen serotypes were identified in carcasses, predominantly O8:H19 (n = 5), O91:H21 (n = 4) and O174:H21 (n = 4). On the other hand, 114 strains isolated from cuts and 45 strains isolated from trimmings were grouped into 37 and 22 different serotypes, respectively. The most prevalent isolated serotypes were O174:H21 (n = 18), O185:H7 (n = 17), O178:H19 (n = 13) and O8:H19 (n = 12). Non-O157 STEC serotypes isolated from all pools of samples are shown in S1 Table.

### Genotypic characterization

Non-O157 STEC isolates presented different variants of the Shiga toxin gene: *stx*_2a_ (n = 59; 29.5%), *stx*_2c(vh-b)_ (n = 51; 25.5%), *stx*_1a_/*stx*_2a_ (n = 26; 13.0%), *stx*_2c(vh-a)_ (n = 18; 9.0%), *stx*_2a_*/stx*_2c(vh-b)_ (n = 15; 7.5%), *stx*_1a_/*stx*_2c(vh-b)_ (n = 15; 7.5%), *stx*_1a_ (n = 6; 3.0%), *stx*_2b_ (n = 4; 2.0%), *stx*_1a_/*stx*_2a_/*stx*_2c(vh-b)_ (n = 3; 1.5%), *stx*_2NT_ (n = 2; 1.0%), *stx*_1a_/*stx*_2c(vh-a)_ (n = 1; 0.5%) and *stx*_1a_/*stx*_2c(vh-b)_ (n = 1; 0.5%). Virulence genes *ehx*A (n = 123; 61.5%) and *saa* (n = 106; 53.0%) were also detected in the strains. However, *eae* and *aggR*+*aaiC* were detected only in one strain each.

Twenty different virulence genotypes were established given the combinations of virulence factors. The most prevalent genotype was *stx*_2c(vh-b)_ (35 strains), followed by *stx*_2a_/*saa*/*ehxA* (32 strains) and *stx*_1a_/*stx*_2a_/*saa/ehxA* (24 strains). O103:H26 STEC carrying the *ehxA* gene was isolated from one (0.5%) pool of striploin. In addition, one strain *saa* and *ehx*A-positive O178:H19 STEC was isolated from one (0.5%) pool of trimmings. Non-O157 STEC genotypes isolated from all pools of samples are shown in S2 Table.

Only one STEC strain (O103:H21) was *eae* positive (0.5%). The *ehxA* gene was carried by 123 (61.5%) of the studied strains. From these *ehxA-*positive strains, 106 were also *saa*-positive. One *ehxA*-negative strain harbored the *saa* gene (O185:H7). As expected, the *eae*-positive strain harbored the *efa1* gene, while all were *toxB*-negative. The most prevalent putative adhesin was that encoded by the *iha* gene, where 164 strains were positive (82%). Genetic markers related to *subAB*, *cdt-V* and *astA* toxins were present in 89 (44.5%), 12 (6%) and 11 (5.5%) strains, respectively. Interestingly, eight *cdt-V*-positive strains were *subAB*-positive, and the other four, reported for the first time in non-O157 STEC strains, were *subAB*-negative. Those strains belonged to serotypes O91:H21 (n = 2) and O48:H7 (n = 1), and one strain was ONT. The *astA*-positive strains belonged to serotypes O174:H21 (n = 7), O171:H2 (n = 1) and O113:H21 (n = 2), and two were ONT. *astA*-positive strains could not be subtyped by PFGE because of bacterial lysis. One *aggR* and *aaiC*-positive strain (O178:H19) was also found.

### PFGE characterization of non-O157 STEC strains

The clonal relatedness of 168 non-O157 STEC strains was established by PFGE of genomic DNA after digestion with *Xba*I (S1 Fig). Thirty-two isolates were excluded from the *Xba*I-PFGE analysis because of bacterial lysis. PFGE analysis showed 144 different patterns with 54.3% similarity and 43 strains grouped in 18 clusters (I to XVIII, 2–4 strains each and 100% homology) (Table 5). Unique patterns were observed in 125 strains.

**Table 5:** *Xba*I-PFGE patterns, serotypes, genotypes, abattoir source and beef sample type of non-O157 STEC strains isolated from the eighteen clusters.

Cluster	*Xba*I-PFGE pattern	Serotype	Genotype	Abattoir	Sample Type	No of isolates
I	3	O185:H7	*stx*_2c(vh-b)_	E	L, HR	2
II	6	O174:H28	*stx*_2a_ + *stx*_2c(vh-b),_ *ehxA*, *saa*	F	S, HR	2
III	10	O185:H7	*stx*_2c(vh-b)_	G	L, HR	2
IV	18	O185:H7	*stx*_2c(vh-b)_	A, E	T	2
V	22	O185:H7	*stx*_2c(vh-a)_	D	L, HR	2
VI	24	O39:H49	*stx*_2a_ + *stx*_2c(vh-b),_ *ehxA*, *saa*	D	S, HR	2
VII	26	O39:H49	*stx*_2a,_ *ehxA*, *saa*	B	S, T	3
				D	HR	
			*stx*_2a_	B	L	1
VIII	29	O39:H49	*stx*_2a,_ *ehxA*, *saa*	G	S, HR	2
IX	32	O113:H21	*stx*_1a,_ *stx*_2a,_*ehxA*, *saa*	D	L, S, HR	3
X	33	O113:H21	*stx*_2a,_*ehxA*, *saa*	B	L	2
				G	T	
XI	58	O130:H11	*stx*_1a,_ *stx*_2c(vh-b),_ *ehxA*, *saa*	D	S, HR	2
XII	69	O178:H19	*stx*_2c(vh-b)_	F	S, HR	2
XIII	70	O22:H8	*stx*_2c(vh-b)_	D	S, T	2
XIV	94	O174:H21	*stx*_2a_	B	L, S, HR	3
XV	98	O164:H8	*stx*_1a,_ *ehxA*, *saa*	A	S	2
				E	HR	
XVI	101	O8:H16	*stx*_1a_, *stx*_2a_, *ehxA*, *saa*	C	S, HR	3
				D	T	
			*stx*_1a_, *ehxA*, *saa*	E	T	1
XVII	105	ONT:H18	*stx*_2a_	G	L, S, HR	3
XVIII	123	O8:H19	*stx*_2a_	A, F	C	2

C: carcass; L: loin; S: striploin; HR: heart of rump; T: trimmings

Strains with identical *Xba*I-PFGE profile were isolated from different samples of the same abattoir on the same sampling date (clusters I, II, III, V, VI, VIII, IX, XI, XII, XIII, XIV, XVII). Strains of each cluster also presented the same serotype and identical genotypic characterization and *Bln*I-PFGE profile, excepting isolates from cluster XVIII that showed the same genotype but three bands of difference in the *Bln*I-PFGE profile and 84.0% similarity. Identical strains were isolated from loin and heart of rump of three clusters (I, III and V), from striploin and heart of rump of five clusters (II, VI, VIII, XI and XII), and from loin, striploin and heart of rump of three other clusters (IX, XIV and XVIII). This cross-contamination among different cuts within an abattoir occurred in five of the eight abattoirs analyzed (once in abattoirs B and E, twice in F, three times in G and five times in D).

Strains from more than one abattoir were grouped into six clusters. Cluster IV included two strains from abattoirs A and E isolated 76 days apart (96% similarity by *Bln*I-PFGE and only one band of difference). Cluster VII included four strains, three isolated on the same day from different samples of abattoir B and one strain isolated 83 days apart from abattoir D; one of the strains from abattoir B had one band of difference, 92.8% similarity by *Bln*I-PFGE and different genotype. Cluster X included two strains from abattoirs B and G sampled 60 days apart. Cluster XV comprised of two strains from abattoirs A and E sampled 61 days apart. Cluster XVI contained two strains isolated from abattoir C and one from abbatoir D sampled on the same date, and one from abattoir E sampled 30 days apart; the strain isolated from abattoir D had identical genotype but one band of difference in the *Bln*I-PFGE profile (85.6% similarity) and the strain from abattoir E had the same *Bln*I-PFGE profile as the others, but it lacked the *stx*_2_ gene. Cluster XVIII comprised two strains from abattoirs A and F isolated 56 days apart.

## Discussion

In this study, we observed that the prevalence of non-O157 STEC in beef carcasses, cuts and trimmings from eight Argentinean abattoirs was low (6.0%). The prevalence of STEC in carcasses reported here was 5.8%, even lower than the 9% previously reported in Argentinean abattoirs using the same isolation methodology [15], possibly due to the application of SENASA intervention measures since 2013 in order to reduce these bacteria[30]. In Poland, STEC prevalence in carcasses was 3.0%, including six “rare” non-O157 serotypes [31], one of which (O185:H7) was identified in the present study. In an Irish slaughterhouse, 27.0% of carcasses was reported positive for non-O157 STEC and four non-O157 STEC serotypes were isolated [32], but none of them was isolated in our study. In US processing plants, STEC prevalence was 53.9% and 8.3% in pre- and post-intervention carcasses, respectively [33], whereas Barkocy-Gallagher et al. [34] reported 8.9% of non-O157 STEC strain from post-intervention carcasses.

Currently, some serotypes are considered as adulterant in raw beef manufacturing trimmings by USDA (2011) [35], the top six being O26, O45, O111, O103, O121 and O145. Thus, different studies have been performed to determine their prevalence in carcasses. Thomas et al. [36] screened for STEC O26, O103, O111 and O145 in Irish carcasses, reporting 5.5% and 2.2% STEC O103 prevalence in pre-evisceration and post-wash carcasses, respectively. In the US, Stromberg et al. [37] [38] reported 4% and 7% of prevalence corresponding to the top six serogroups in pre-intervention carcasses of feedlot cattle and cully dairy cows, respectively. In New Zealand, STEC O26 prevalence in pre- and post-intervention carcasses was 17.2% and 10.6%, respectively [39]. Using a different methodology, Bosilevac et al. [40] recently screened for O5, O84, O118 and O177 besides the top six serogroups in carcasses, reporting a total prevalence of 38.7%. In our study, however, we did not find O26, O45, O103, O111, O121, O145, or any of the serogroups described by Bosilevac et al. [40].

Very few studies have reported the prevalence of all non-O157 STEC serotypes on cuts and trimming. In the present study, 5.8% and 7.0% of pools from cuts and trimmings, respectively, were positive for non-O157 STEC in the eight studied abattoirs. In Namibia, the prevalence of *stx* and serogroup genes (O26, O45, O103, O111, O121 or O145) in beef trimming samples screened by PCR was 17.7% and 11.3%, respectively [41]. In Iran, the prevalence of top six serogroups and STEC O91, O113 and O128 from retail beef was 13.5% [42]. In the US, non-O157 STEC prevalence was 9.4% in retail beef [43] and 6.6, 1.8, 4.6 and 20.3% in domestic and imported beef trimmings from New Zealand, Australia and Uruguay, respectively [44]. Our findings were similar to those reported by Bosilevac et al. [44] in beef trimmings, and also agree with the previously reported low prevalence (0.6%) of top six STEC in Argentina identifying only one O26 STEC from retail raw ground beef [45].

Some of the serotypes found in this study (O8:H19, O91:H21, O113:H21, O130:H11, O174:H21 and O178:H19) have been previously isolated from cattle, food and the environment in Argentina [46]. O8:H19, O174:H21 and O178:H19 were the most frequently found; they were isolated from all types of sources and associated with at least one HUS case in Argentina [45, 47–49]. However, none were associated with HUS during our study period according the Argentinean surveillance system [50]. Serotypes O130:H11 and O185:H7 were also isolated from all types of sources, but they were not associated with HUS. *E*. *coli* O130:H11 and O178:H19 were the most prevalent serotypes isolated from dairy cows [51] and had been previously identified in beef abattoirs [15]. Most non-O157 STEC serotypes isolated from bovine carcasses and meat in the present work have already been described [45, 52–55], except for O8:H7, O20:H7, O41:H7, O48:H7, O73:H[41, NM], O74:H42, O83:H25, O84:H7, O103:H[26, 42], O141:NM, O149:H[8, 16], O154:H19, O163:H[28, 46], O181:H14, O183:H18, O185:H[21, NM] and ONT:H[18, 41, 66]. Although their pathogenic potential on the basis of the non-O157 STEC serotypes isolated from HUS cases has been assessed, the European Food Safety Authority (EFSA) moved away from such classification due to the difficulty of predicting the emergence of novel pathogenic STEC types considering only the *eae* gene or a restricted panel of serogroups [7].

PFGE analysis showed clonal relatedness among strains isolated from cuts and/or trimmings of the same abattoirs (B, D, E, F and G), probably due to cross contamination in the deboning process. Cross contamination with STEC among carcasses, trimmings and the environment has been previously described [56, 57]. Five clusters grouped clonal STEC strains obtained from at least two types of samples. In a previous study developed in Argentina, clonal strains were isolated from carcasses from different exporting abattoirs on different sampling dates [13]. Despite in the present study a greater number of samples was analyzed, STEC strain clones were not persistent. From six clusters grouping strains from more than one abattoir, five (IV, VII, X, XV, XVI) included strains from cuts and trimmings, and one (XVIII) included strains from carcasses. The presence of clonal strains in samples from different abattoirs could be related to the common origin of cattle [15].

Food safety programs often impose conditions on imported foodstuffs to protect the population. In this sense, screening for all STEC, regardless of the risk to consumers, can divert the attention to low or unknown risk strains, precluding the screening for high-risk strains. Also, limitations in laboratory procedures, such as the discordant results across protocols and the availability of methods only for 10 non-O157 STEC serotypes, are some of the problems affecting the application of zero-tolerance criteria [37, 58]. Bosilevac et al. [40], besides the top six STEC serotypes, searched for another three (O5, O84, O118) identified by the Centers for Disease Control and Prevention in their non-O157 STEC prevalence report, and one (O177) associated with HUS. In the present study, we focused on the isolation of all 1,152 non-O157 STEC and isolated only serogroup O103 from the top six [8].

Karmali *et al*. [59] classified STEC strains into seropathotypes from A to E, according to incidence and association with HUS and outbreaks. Serotypes that associated with severe illness corresponded to seropathotypes A to C. From the 200 strains isolated in this work, one O103:H21 corresponded with serophatotype B, whereas seven O91:H21 and nine O113:H21 strains associated with serophatotype C. Although this classification was of great value to define pathogenic STEC serotypes in human infection and strains isolated from animals [60, 61], the progressive increase of non-O157 STEC cases and outbreaks has drawn attention to its usefulness [7, 62].

The genotypic profiles of STEC strains isolated from carcasses, meat cuts and trimmings in the present study have been previously reported in Argentina [15]. One hundred and six (53.0%) strains harbored simultaneously *saa* and *ehxA*, both genes related with strain virulence[63]. According to the Joint FAO/WHO Expert Meetings on Microbiological Risk Assessment (JEMRA) [58], the presence of one of the *stx*_1_, *stx*_2_, *eae*, *aggR* and/or *aaiC* genes is insufficient to predict the likelihood to cause severe illness. Therefore, the combination of these genes should be used to predict health risk. In agreement with EFSA[7], JEMRA sentenced that *stx*_2_ and *eae* or *stx*_2_ and *aggR*/*aaiC* are reliable predictors of high risk [58]. Accordingly, one O113:H21 (*stx*_1a_/*stx*_2a_/*eae*/*ehxA*) strain isolated from loin and one O178:H19 (*stx*_1a_/*stx*_2a_/*ehxA*/*aggR*/*aaiC*) isolated from trimmings in abattoir D presented a genotypic profile with higher potential risk of causing severe illness. Despite their potential risk according to JEMRA [58], strains with this serotype and genotypic profile were not reported by the Argentinean surveillance system during the study period [50].

Historically, meat has been associated with HUS [63, 64] because cattle are the main reservoir of STEC. In this paper, 200 non-O157 STEC strains were isolated from abattoirs, but none had the sero-genotype profile of non-O157 STEC strains reported by the surveillance system during the same study period [50]. The underreporting of HUS cases, the scarce number of cases with confirmed source and the fact that the illness-causing strain is seldom found in foods have been recognized as problems on the epidemiology of HUS and STEC[58]. In Argentina, despite the endemic character of HUS, only four cases were associated with food consumption between 2002 and 2015, all associated with STEC O157:H7 *stx*_2_/*eae*/*ehxA* [9, 50]. It is very important to identify non-O157 STEC associated with diseases to establish links between sources and human cases, and conduct food surveillance directed towards these strains. Outbreak studies should be reinforced in order to identify the source of contamination and its association with the food production chain, including human-human infection.

Zero-tolerance criteria are applied for imported meat in the EU since 2012 [65]. During 2013, approximately 90 border rejection cases were reported for chilled beef coming from different countries [13]. Since fecal contamination of meat during processing at abattoirs is unavoidable [66], zero tolerance intervention measures should be applied to all STEC in meat, together with GMP and HACCP [39]. Arthur et al. [33], in a study of large processing plants in the US, demonstrated that STEC prevalence dramatically diminished (58.3%–8.3%) in carcasses previously treated with different antimicrobial intervention strategies such as steam vacuuming, hot water washing, organic acid washing and steam pasteurization. Washing with acid could be useful in order to diminish STEC prevalence in meat[67]. Kalchayanand et al. [68] reported that reduction of non-O157 STEC by some antimicrobial interventions was at least as efficient as for O157. In Argentina, SENASA authorized the application of steam vacuum and diluted organic acids in carcass surfaces since 2013 [30]. In the EU, only lactic acid was approved for use on beef carcasses as an antimicrobial intervention [69]. However, USDA permits the use of several substances and has approved on-line reprocessing and off-line reprocessing antimicrobial intervention systems since March 14, 2017, which are commonly applied to beef carcasses in the US [35]. The application of single or multiple intervention measures on carcasses and cuts would help diminish non-O157 STEC prevalence.

## Conclusions

STEC monitoring in foods should be developed for a valid purpose and should be commodity-specific [58]. Our results showed non-O157 STEC prevalence and strain profiles in beef from Argentinean abattoirs through characterization and genotypic analysis. The prevalence of non-O157 STEC in carcasses reported here was lower than previously reported in Argentina. This is the first study simultaneously screening for non-O157 STEC in cuts and trimmings at eight Argentinean abattoirs. None of the non-O157 STEC strains isolated corresponded with the serotype and virulence profiles isolated from human cases in Argentina in the same study period [50]. However, the risk of meat contaminated with non-O157 STEC to consumers cannot be determined through the mere analysis of their prevalence in beef and the genotypic profile of strains. Risk analysis of every food category considering the habits of consumer groups and the geographical and temporal relationship with human and food strains is necessary to determine the microbiological criteria for each foodstuff. In this sense, it is very important to have a stringent monitoring system.

Besides GMP and HACCP, the application of intervention measures such as washing carcasses and cuts with acid is necessary to meet the zero-tolerance criteria for non-O157 STEC from beef. Collaborative efforts for risk assessment, management and communication are extremely necessary to give an insight into the real clinical implications of virulence genes and allow the classification of STEC strains more efficiently according to risk in order to improve the safety of foodstuffs.

## Supporting information

S1 Table Genotypes (*stx*, *eae*, *ehxA*, *aggR*+*aaiC*) of non-O157 STEC strains isolated from beef carcasses, anatomical cuts and trimmings from Argentinean abattoirs. (PDF)

S2 Table Genotypes (*stx*, *eae*, *ehxA*, *aggR*+*aaiC*) of non-O157 STEC strains isolated from beef carcasses, anatomical cuts and trimmings from Argentinean abattoirs. (PDF)

S1 Fig *Xba*I-PFGE UPGMA dendrogram. Sampling type and stage, serotypes and genotypes of 168 non-O157 STEC strains isolated from abbatoirs.
(PDF)
