# Supplementary material for: Isolation and characterization of non-O157 Shiga toxin-producing Escherichia coli from beef carcasses, cuts and trimmings of abattoirs in Argentina
Source: PLoS One. 2017 Aug 22;12(8):e0183248. doi: 10.1371/journal.pone.0183248 (PMC5568767; doi:10.1371/journal.pone.0183248)
Supplement: S1 Table — (PDF) [file pone.0183248.s001.pdf]

S1. Serotypes of non-O157 STEC strains isolated from beef carcasses, anatomical cuts and trimmings from Argentinean abattoirs.

Serotype	Carcasses	Loin	Striploin	Heart of rump	Trimmings	TOTAL
O8:H7	1					**1**
O8:H16*	1		1	1	2	**5**
O8:H19*	5	6	1	3	2	**17**
O15:H27*		1	1			**2**
O20:H7	1	2		1		**4**
O22:H8	1		2		3	**6**
O22:H16					1	**1**
O39:H49		1	3	4	3	**11**
O41:H7				1		**1**
O46:H38				1		**1**
O48:H7		2		1		**3**
O73:H41			1			**1**
O73:NM				1		**1**
O74:H42			1			**1**
O82:H8	1					**1**
O83:H25	1					**1**
O84:H7			1			**1**
O88:H25					1	**1**
O91:H21*	4	1	1		1	**7**
O103:H21*			1			**1**
O103:H26					1	**1**
O103:H42				1		**1**
O113:H21*		2	3	1	3	**9**
O130:H11	3	1	1	2	5	**12**
O130:H21			1		1	**2**
O141:H49	1	1		1		**3**
O149:H8	2					**2**
O149:H16					1	**1**
O154:H19					1	**1**
O163:H19	2			1		**3**
O163:H28				1		**1**
O163:H46					1	**1**
O164:H8			1	1		**2**
O171:H2			1			**1**
O171:NM					1	**1**
O174:H21*	4	3	4	6	5	**22**
O174:H28*		1	1	1		**3**
O178:H19*	2	1	5	4	3	**15**
O179:H8	2	2				**4**

O181:H4	1					**1**
O183:H18		1				**1**
O185:H7	2	3	2	8	4	**19**
O185:H21	1					**1**
O185:NM				1		**1**
ONT:H8			1		1	**2**
ONT:H18		1	1	1		**3**
ONT:H19	1			1		**2**
ONT:H21	2		3	1	3	**9**
ONT:H25	1					**1**
ONT:H38				1		**1**
ONT:H41	1					**1**
ONT:H46		2		1	1	**4**
ONT:H66	1					**1**
ONT:HNT[**]					1	**1**
**TOTAL**	**41**	**31**	**37**	**46**	**45**	**200**

[*] non-O157 STEC serotypes associated with at least one HUS case in Argentina.

[**] non-O157 STEC serotypes associated with HUS in Argentina during 2011-2015 (BIV Nº329)
