# Supplementary material for: Isolation and characterization of non-O157 Shiga toxin-producing Escherichia coli from beef carcasses, cuts and trimmings of abattoirs in Argentina
Source: PLoS One. 2017 Aug 22;12(8):e0183248. doi: 10.1371/journal.pone.0183248 (PMC5568767; doi:10.1371/journal.pone.0183248)
Supplement: S2 Table — (PDF) [file pone.0183248.s002.pdf]

**S2.** Genotypes (*stx*, *eae*, *ehxA*, *aggR+aaiC*) of non-O157 STEC strains isolated from beef carcasses, anatomical cuts and trimmings from Argentinean abattoirs.

| Genotype | Carcasses | Loin | Striploin | Heart of rump | Trimmings | TOTAL |
|---|---|---|---|---|---|---|
| $stx_{1a}$/*saa/ehxA* | | 3 | 1 | 1 | 1 | **6** |
| $stx_{2a}$ | 2 | 3 | 3 | 3 | 3 | **14** |
| $stx_{2a}$/*ehxA* | 5 | 4 | 1 | 2 | 1 | **13** |
| $stx_{2a}$/*saa/ehxA* | 5 | 9 | 4 | 6 | 8 | **32** |
| $stx_{2a}$/$stx_{2c(vh\text{-}b)}$ | | | 1 | | 1 | **2** |
| $stx_{2a}$/$stx_{2c(vh\text{-}b)}$/*saa/ehxA* | 3 | | 3 | 4 | 3 | **13** |
| $stx_{2b}$ | | | 1 | 1 | 2 | **4** |
| $stx_{2c(vh\text{-}a)}$ | 2 | 3 | 4 | 7 | 1 | **17** |
| $stx_{2c(vh\text{-}a)\,)}$/*saa* | | | | 1 | | **1** |
| $stx_{2c(vh\text{-}b)}$ | 8 | 4 | 7 | 6 | 10 | **35** |
| $stx_{2c(vh\text{-}b)}$/*ehxA* | 2 | | 1 | | 1 | **4** |
| $stx_{2c(vh\text{-}b)}$/*saa/ehxA* | 3 | 1 | 1 | 5 | 2 | **12** |
| $stx_{2NT}$ | 1 | | 1 | | | **2** |
| $stx_{1a}$/$stx_{2c(vh\text{-}a)}$ | | | 1 | | | **1** |
| $stx_{1a}$/$stx_{2c(vh\text{-}b)}$ | | | 1 | | | **1** |
| $stx_{1a}$/$stx_{2a}$/*eae/ehxA* | | | 1 | | | **1** |
| $stx_{1a}$/$stx_{2a}$/*saa/ehxA* | 5 | 2 | 2 | 8 | 7 | **24** |
| $stx_{1a}$/$stx_{2a}$/*saa/ehxA/aggR+aaiC* | | | | | 1 | **1** |
| $stx_{1a}$/$stx_{2a}$/$stx_{2c(vh\text{-}b)}$/*saa/ehxA* | | 2 | | 1 | | **3** |
| $stx_{1a}$/$stx_{2c(vh\text{-}b)}$/*saa/ehxA* | 5 | | 4 | 1 | 4 | **14** |
| **TOTAL** | **41** | **31** | **37** | **46** | **45** | **200** |
