# Supplementary material for: Isolation and characterization of non-O157 Shiga toxin-producing Escherichia coli from beef carcasses, cuts and trimmings of abattoirs in Argentina
Source: PLoS One. 2017 Aug 22;12(8):e0183248. doi: 10.1371/journal.pone.0183248 (PMC5568767; doi:10.1371/journal.pone.0183248)
Supplement: S1 Fig — Sampling type and stage, serotypes and genotypes of 168 non-O157 STEC strains isolated from abbatoirs. (PDF) [file pone.0183248.s003.pdf]

S3. XbaI-PFGE UPGMA dendrogram. Sampling type and stage, serotypes and genotypes of 168 non-O157 STEC strains isolated from abbatoirs.

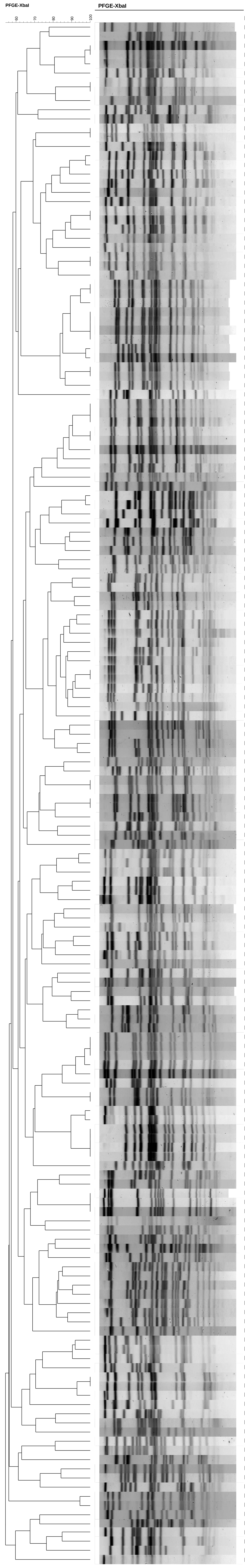

Key	Abattoir	Sample type	Serotype	Genotype
M-C-5-14 (1)	C	Trimmings	O178:H19	ehxA/saa/iha/lpf2-2/subAB/$stx_{2c\ (vh-b)}$
M-G-2-14 (8)	G	Loin	O174:H21	ehxA/saa/iha/lpf2-2/subAB/stx_{2a}
M-E-2-14 (1)	E	Loin	O185:H7	iha/lpf2-2/$stx_{2c\ (vh-b)}$
M-E-4-14 (1)	E	Heart of rump	O185:H7	iha/lpf2-2/$stx_{2c\ (vh-b)}$
M-E-3-14 (1)	E	Striploin	O185:H7	iha/lpf2-2/$stx_{2c\ (vh-b)}$
M-E-4-14 (6)	E	Heart of rump	O185:H7	iha/lpf2-2/$stx_{2c\ (vh-a)}$
M-F-3-14 (2)	F	Striploin	O174:H28	ehxA/saa/iha/lpf2-2/subAB/stx_{2a}+$stx_{2c\ (vh-b)}$
M-F-4-14 (10)	F	Heart of rump	O174:H28	ehxA/saa/iha/lpf2-2/subAB/stx_{2a}+$stx_{2c\ (vh-b)}$
M-F-4-14 (7)	F	Heart of rump	O174:H21	ehxA/saa/iha/lpf2-2/subAB/stx_{2a}+$stx_{2c\ (vh-b)}$
M-A-3-14 (1)	A	Striploin	ONT:H21	ehxA/saa/iha/lpf2-2/stx_{1a}/$stx_{2c\ (vh-b)}$
M-C-5-14 (2)	C	Trimmings	ONT:H21	ehxA/saa/iha/lpf2-2/stx_{1a}/stx_{2a}
M-G-2-14 (1)	G	Loin	O185:H7	iha/lpf2-2/$stx_{2c\ (vh-b)}$
M-G-4-14 (2)	G	Heart of rump	O185:H7	iha/lpf2-2/$stx_{2c\ (vh-b)}$
M-A-4-14 (5)	A	Heart of rump	O185:H7	iha/lpf2-2/$stx_{2c\ (vh-b)}$
M-A-4-14 (3)	A	Heart of rump	O185:H7	saa/lpf2-2/subAB/$stx_{2c\ (vh-a)}$
M-D-3-14 (2)	D	Striploin	O185:H7	iha/lpf2-2/$stx_{2c\ (vh-a)}$
M-E-4-14 (5)	E	Heart of rump	O185:H7	iha/lpf2-2/subAB/cdt-V/$stx_{2c\ (vh-a)}$
M-F-4-14 (9)	F	Heart of rump	O185:H7	iha/lpf2-2/$stx_{2c\ (vh-a)}$
M-F-1-14 (2)	F	Carcass	O185:H7	iha/lpf2-2/$stx_{2c\ (vh-b)}$
M-D-5-14 (8)	D	Trimmings	O185:H7	lpf2-2/$stx_{2c\ (vh-a)}$
M-A-5-14 (1)	A	Trimmings	O185:H7	iha/lpf2-2/$stx_{2c\ (vh-b)}$
M-E-5-14 (7)	E	Trimmings	O185:H7	iha/lpf2-2/$stx_{2c\ (vh-b)}$
M-E-5-14 (6)	E	Trimmings	O185:H7	iha/lpf2-2/$stx_{2c\ (vh-b)}$
M-A-1-14 (2)	A	Carcass	O185:H7	lpf2-2/$stx_{2c\ (vh-b)}$
M-A-4-14 (2)	A	Heart of rump	O185:NM	iha/lpf2-2/subAB/$stx_{2c\ (vh-a)}$
M-D-2-14 (1)	D	Loin	O185:H7	iha/lpf2-2/stx_{2}
M-D-4-14 (1)	D	Heart of rump	O185:H7	iha/lpf2-2/$stx_{2c\ (vh-a)}$
M-D-1-14 (7)	D	Carcass	O185:H21	ehxA/lpf2-2/$stx_{2c\ (vh-b)}$
M-D-3-14 (4)	D	Striploin	O39:H49	ehxA/saa/iha/lpf2-2/subAB/stx_{2a}+$stx_{2c\ (vh-b)}$
M-D-4-14 (4)	D	Heart of rump	O39:H49	ehxA/saa/lpf2-2/subAB/stx_{2a}+$stx_{2c\ (vh-b)}$
M-G-4-14 (9)	G	Heart of rump	O39:H49	ehxA/saa/iha/lpf2-2/subAB/stx_{1a}/stx_{2a}
M-B-2-14 (1)	B	Loin	O39:H49	lpf2-2/stx_{2a}
M-B-3-14 (1)	B	Striploin	O39:H49	ehxA/saa/iha/lpf2-2/subAB/stx_{2a}
M-B-5-14 (4)	B	Trimmings	O39:H49	ehxA/saa/iha/lpf2-2/subAB/cdt-V/stx_{2a}
M-D-4-14 (9)	D	Heart of rump	O39:H49	ehxA/saa/iha/lpf2-2/subAB/stx_{2a}
M-E-5-14 (1)	E	Trimmings	O39:H49	ehxA/saa/iha/lpf2-2/subAB/cdt-V/stx_{2a}
M-G-3-14 (1)	G	Striploin	ONT:H21	ehxA/saa/iha/lpf2-2/subAB/stx_{2a}
M-G-3-14 (4)	G	Striploin	O39:H49	ehxA/saa/iha/lpf2-2/subAB/stx_{2a}
M-G-4-14 (3)	G	Heart of rump	O39:H49	ehxA/saa/iha/lpf2-2/subAB/stx_{2a}
M-G-5-14 (2)	G	Trimmings	O39:H49	ehxA/saa/iha/lpf2-2/subAB/stx_{2a}
M-E-3-14 (3)	E	Striploin	O84:H7	ehxA/saa/iha/lpf2-2/stx_{2a}
M-D-2-14 (5)	D	Loin	O113:H21	ehxA/saa/iha/lpf2-2/subAB/stx_{1a}/stx_{2a}
M-D-3-14 (10)	D	Striploin	O113:H21	ehxA/saa/iha/lpf2-2/subAB/stx_{1a}/stx_{2a}
M-D-4-14 (8)	D	Heart of rump	O113:H21	ehxA/saa/iha/lpf2-2/subAB/stx_{1a}/stx_{2a}
M-B-5-14 (3)	B	Trimmings	O113:H21	ehxA/saa/iha/lpf2-2/subAB/stx_{2a}
M-G-2-14 (7)	G	Loin	O113:H21	ehxA/saa/iha/lpf2-2/subAB/stx_{2a}
M-F-4-14 (1)	F	Heart of rump	ONT:H21	ehxA/saa/iha/lpf2-2/subAB/stx_{2a}+$stx_{2c\ (vh-b)}$
M-E-5-14 (4)	E	Trimmings	O113:H21	ehxA/saa/iha/lpf2-2/subAB/stx_{2a}+$stx_{2c\ (vh-b)}$
M-G-5-14 (5)	G	Trimmings	ONT:H21	ehxA/saa/lpf2-2/subAB/cdt-V/stx_{2a}
M-D-5-14 (3)	D	Trimmings	O178:H19	ehxA/saa/aggR/aaiC/iha/lpf2-2/stx_{1a}/stx_{2a}
M-C-1-14 (2)	C	Carcass	O181:H4	ehxA/saa/iha/lpf2-2/subAB/stx_{1a}/stx_{2a}
M-F-2-14 (3)	F	Loin	O20:H7	iha/lpf2-2/stx2$_{c\ (vh-a)}$
M-F-2-14 (4)	F	Loin	O20:H7	iha/lpf2-2/stx2$_{c\ (vh-a)}$
M-E-4-14 (4)	E	Heart of rump	O20:H7	lpf2-2/subAB/stx2$_{c\ (vh-a)}$
M-E-1-14 (4)	E	Carcass	O20:H7	iha/lpf2-2/$stx_{2c\ (vh-b)}$
M-E-5-14 (3)	E	Trimmings	O22:H8	iha/lpf2-2/stx_{2a}
M-G-3-14 (6)	G	Striploin	O22:H8	iha/lpf2-2/stx_{2a}
M-F-5-14 (1)	F	Trimmings	O22:H8	iha/lpf2-2/stx_{2a}
M-G-3-14 (11)	G	Striploin	O113:H21	iha/lpf2-2/stx_{1a}/$stx_{2c\ (vh-a)}$
M-G-3-14 (7)	G	Striploin	O113:H21	iha/lpf2-2/astA/stx_{1a}/$stx_{2c\ (vh-b)}$
M-D-1-14 (14)	D	Carcass	O130:H11	ehxA/saa/iha/lpf2-2/subAB/stx_{1a}/$stx_{2c\ (vh-b)}$
M-E-5-14 (5)	E	Trimmings	O130:H21	ehxA/saa/iha/lpf2-2/subAB/stx_{1a}/stx_{2a}
M-D-1-14 (10)	D	Carcass	O149:H8	ehxA/saa/iha/lpf2-2/subAB/stx_{2a}/$stx_{2c\ (vh-b)}$
M-D-1-14 (8)	D	Carcass	O149:H8	ehxA/saa/iha/lpf2-2/subAB/$stx_{2c\ (vh-b)}$
M-D-5-14 (6)	D	Trimmings	O130:H11	ehxA/saa/iha/lpf2-2/subAB/stx_{1a}/stx_{2a}
M-G-3-14 (9)	G	Striploin	O130:H21	ehxA/saa/lpf2-2/subAB/stx_{1a}/$stx_{2c\ (vh-b)}$
M-G-5-14 (3)	G	Trimmings	O130:H11	ehxA/saa/lpf2-2/subAB/stx_{1a}/stx_{2a}
M-G-4-14 (1)	G	Heart of rump	O130:H11	ehxA/saa/iha/lpf2-2/subAB/stx_{1a}/stx_{2a}
M-B-5-14 (2)	B	Trimmings	O130:H11	ehxA/saa/iha/lpf2-2-/subAB/stx_{1a}/$stx_{2c\ (vh-b)}$
M-G-2-14 (3)	G	Loin	O130:H11	ehxA/saa/iha/lpf2-2/subAB/stx_{1a}/stx_{2a}+$stx_{2c\ (vh-b)}$
M-D-3-14 (6)	D	Striploin	O130:H11	ehxA/saa/iha/lpf2-2/subAB/stx_{1a}/$stx_{2c\ (vh-b)}$
M-D-4-14 (5)	D	Heart of rump	O130:H11	ehxA/saa/iha/lpf2-2/subAB/stx_{1a}/$stx_{2c\ (vh-b)}$
M-C-1-14 (1)	C	Carcass	O130:H11	ehxA/saa/iha/lpf2-2/subAB/stx_{1a}/$stx_{2c\ (vh-b)}$
M-D-1-14 (19)	D	Carcass	O130:H11	ehxA/saa/lpf2-2/subAB/stx_{1a}/$stx_{2c\ (vh-b)}$
M-F-5-14 (2)	F	Trimmings	O130:H11	ehxA/saa/iha/lpf2-2/subAB/stx_{1a}/$stx_{2c\ (vh-b)}$
M-G-5-14 (1)	G	Trimmings	O130:H11	ehxA/saa/iha/lpf2-2/subAB/stx_{1a}/$stx_{2c\ (vh-b)}$
M-A-2-14 (2)	A	Loin	O179:H8	ehxA/saa/iha/lpf2-2/subAB/stx_{2a}
M-D-1-14 (5)	D	Carcass	O179:H8	ehxA/saa/iha/lpf2-2/subAB/stx_{1a}/stx_{2a}
M-D-1-14 (4)	D	Carcass	O179:H8	ehxA/saa/iha/lpf2-2/subAB/stx_{2a}+$stx_{2c\ (vh-b)}$
M-D-2-14 (3)	D	Loin	O179:H8	ehxA/saa/iha/lpf2-2/subAB/cdt-V/stx_{2a}
M-E-4-14 (3)	E	Heart of rump	O178:H19	iha/lpf2-2/subAB/$stx_{2c\ (vh-a)}$
M-F-4-14 (5)	F	Heart of rump	ONT:H19	ehxA/saa/iha/lpf2-2/subAB/$stx_{2c\ (vh-b)}$
M-F-3-14 (1)	F	Striploin	O178:H19	iha/lpf2-2/$stx_{2c\ (vh-b)}$
M-F-4-14 (3)	F	Heart of rump	O178:H19	iha/lpf2-2/$stx_{2c\ (vh-b)}$
M-D-3-14 (7)	D	Striploin	O22:H8	lpf2-2/$stx_{2c\ (vh-b)}$
M-D-5-14 (5)	D	Trimmings	O22:H8	lpf2-2/$stx_{2c\ (vh-b)}$
M-D-1-14 (12)	D	Carcass	O22:H8	ehxA/iha/lpf2-2/$stx_{2c\ (vh-b)}$
M-G-2-14 (2)	G	Loin	O174:H28	ehxA/saa/iha/lpf2-2/subAB/stx_{2a}
M-G-5-14 (8)	G	Trimmings	O88:H25	ehxA/saa/lpf2-2/stx_{2a}
M-E-1-14 (1)	E	Carcass	O82:H8	ehxA/saa/iha/lpf2-2/stx1a/$stx_{2c\ (vh-b)}$
M-D-1-14 (15)	D	Carcass	O141:H49	ehxA/saa/iha/lpf2-2/subAB/stx_{2a}
M-G-2-14 (6)	G	Loin	O141:H49	ehxA/saa/iha/lpf2-2/stx_{1a}
M-F-4-14 (4)	F	Heart of rump	O141:H49	ehxA/saa/lpf2-2/subAB/stx_{2a}
M-D-2-14 (2)	D	Loin	O48:H7	ehxA/saa/iha/lpf2-2/stx_{2a}
M-G-4-14 (8)	G	Heart of rump	O48:H7	ehxA/saa/iha/lpf2-2/stx_{1a}/stx_{2a}
M-E-2-14 (3)	E	Loin	O48:H7	ehxA/saa/iha/lpf2-2/stx_{2a}
M-F-1-14 (5)	F	Carcass	O178:H19	iha/lpf2-2/subAB/$stx_{2c\ (vh-b)}$
M-G-3-14 (3)	G	Striploin	O178:H19	iha/lpf2-2/$stx_{2c\ (vh-a)}$
M-D-4-14 (10)	D	Heart of rump	O174:H21	iha/lpf2-2/stx_{2b}
M-F-5-14 (3)	F	Trimmings	O178:H19	ehxA/saa/iha/lpf2-2/subAB/stx_{1a}/stx_{2a}
M-G-2-14 (10)	G	Loin	O178:H19	ehxA/saa/iha/lpf2-2/subAB/stx_{1a}/stx_{2a}
M-G-4-14 (6)	G	Heart of rump	O178:H19	ehxA/saa/subAB/$stx_{2c\ (vh-b)}$
M-D-4-14 (2)	D	Heart of rump	O178:H19	ehxA/saa/iha/lpf2-2/subAB/stx_{2a}
M-C-1-14 (3)	C	Carcass	ONT:H19	ehxA/saa/lpf2-2/subAB/$stx_{2c\ (vh-b)}$
M-D-5-14 (7)	D	Trimmings	O154:H19	ehxA/saa/iha/lpf2-2/subAB/stx_{2a}
M-D-1-14 (11)	D	Carcass	O178:H19	iha/lpf2-2/stx_{2NT}
M-D-3-14 (9)	D	Striploin	O178:H19	iha/lpf2-2/stx_{2NT}
M-B-5-14 (6)	B	Trimmings	O103:H26	ehxA/saa/lpf2-2/stx_{1a}/$stx_{2c\ (vh-b)}$
M-G-3-14 (5)	G	Striploin	O74:H42	ehxA/saa/iha/lpf2-2/stx_{1a}/$stx_{2c\ (vh-b)}$
M-C-4-14 (2)	C	Heart of rump	O103:H42	ehxA/saa/iha/lpf2-2/stx_{1a}/stx_{2a}+$stx_{2c\ (vh-b)}$
M-B-2-14 (2)	B	Loin	O174:H21	iha/lpf2-2/stx_{2a}
M-B-3-14 (2)	B	Striploin	O174:H21	iha/lpf2-2/stx_{2a}
M-B-4-14 (3)	B	Heart of rump	O174:H21	iha/lpf2-2/stx_{2a}
M-B-4-14 (2)	B	Heart of rump	O174:H21	iha/lpf2-2/stx_{2a}
M-B-5-14 (5)	B	Trimmings	ONT:HNT	iha/lpf2-2/stx_{2a}
M-C-1-14 (7)	C	Carcass	O91:H21	ehxA/saa/lpf2-2/subAB/cdt-V/stx_{2a}
M-A-3-14 (2)	A	Striploin	O164:H8	ehxA/saa/iha/lpf2-2/stx_{1a}
M-E-4-14 (7)	E	Heart of rump	O164:H8	ehxA/saa/iha/lpf2-2/stx_{1a}
M-C-2-14 (2)	C	Loin	O8:H19	ehxA/saa/iha/lpf2-2/stx_{1a}
M-D-1-14 (20)	D	Carcass	O8:H16	ehxA/saa/iha/lpf2-2/stx1a/$stx_{2c\ (vh-b)}$
M-C-3-14 (1)	C	Striploin	O8:H16	ehxA/saa/iha/lpf2-2/stx_{1a}/stx_{2a}
M-C-4-14 (1)	C	Heart of rump	O8:H16	ehxA/saa/iha/lpf2-2/stx_{1a}/stx_{2a}
M-D-5-14 (1)	D	Trimmings	O8:H16	ehxA/saa/iha/lpf2-2/stx_{1a}/stx_{2a}
M-E-5-14 (2)	E	Trimmings	O8:H16	ehxA/saa/iha/lpf2-2/stx_{1a}
M-D-4-14 (3)	D	Heart of rump	O41:H7	ehxA/saa/iha/lpf2-2/subAB/$stx_{2c\ (vh-b)}$
M-D-1-14 (16)	D	Carcass	ONT:H21	ehxA/saa/iha/subAB/stx_{1a}/stx_{2a}
M-G-4-14 (4)	G	Heart of rump	O73:NM	ehxA/saa/iha/lpf2-2/subAB/$stx_{2c\ (vh-b)}$
M-G-2-14 (5)	G	Loin	ONT:H18	lpf2-2/subAB/cdt-V/stx_{2a}
M-G-3-14 (8)	G	Striploin	ONT:H18	lpf2-2/subAB/cdt-V/stx_{2a}
M-G-4-14 (5)	G	Heart of rump	ONT:H18	lpf2-2/stx_{2a}
M-D-3-14 (3)	D	Striploin	O178:H19	iha/lpf2-2/$stx_{2c\ (vh-b)}$
M-E-3-14 (2)	E	Striploin	O178:H19	iha/lpf2-2/$stx_{2c\ (vh-b)}$
M-C-5-14 (3)	C	Trimmings	O22:H16	iha/lpf2-2/$stx_{2c\ (vh-b)}$
M-D-1-14 (1)	D	Carcass	ONT:H41	lpf2-2-/stx_{2a}
M-B-3-14 (3)	B	Striploin	O73:H41	ehxA/saa/iha/lpf2-2/subAB/cdt-V/$stx_{2c\ (vh-b)}$
M-D-4-14 (6)	D	Heart of rump	ONT:H46	ehxA/saa/iha/lpf2-2/subAB/stx_{2a}
M-H-5-14 (1)	H	Trimmings	O163:H46	ehxA/saa/lpf2-2/subAB/stx_{2a}
M-G-2-14 (9)	G	Loin	ONT:H46	ehxA/saa/iha/lpf2-2/subAB/$stx_{2c\ (vh-b)}$
M-G-4-14 (10)	G	Heart of rump	O163:H28	ehxA/saa/iha/lpf2-2/subAB/stx_{2a}
M-E-2-14 (2)	E	Loin	ONT:H46	ehxA/saa/iha/lpf2-2/subAB/stx_{2a}
M-E-1-14 (2)	E	Carcass	ONT:H66	ehxA/saa/iha/lpf2-2/subAB/stx_{2a}
M-G-5-14 (7)	G	Trimmings	ONT:H46	ehxA/saa/iha/lpf2-2/subAB/stx_{2a}+$stx_{2c\ (vh-b)}$
M-D-3-14 (5)	D	Striploin	O15:H27	iha/lpf2-2/subAB/$stx_{2c\ (vh-b)}$
M-A-2-14 (1)	A	Loin	O8:H19	ehxA/saa/iha/lpf2-2/stx_{1a}/stx_{2a}+$stx_{2c\ (vh-b)}$
M-A-5-14 (4)	A	Trimmings	O8:H19	ehxA/saa/iha/lpf2-2/stx_{1a}/stx_{2a}
M-D-4-14 (7)	D	Heart of rump	O8:H19	ehxA/saa/iha/lpf2-2/stx_{1a}/stx_{2a}
M-G-2-14 (4)	G	Loin	O91:H21	ehxA/saa/iha/lpf2-2/cdt-V/stx_{2a}
M-A-1-14 (1)	A	Carcass	O8:H19	ehxA/lpf2-2/stx_{2a}
M-F-1-14 (4)	F	Carcass	O8:H19	ehxA/lpf2-2/stx_{2a}
M-F-2-14 (2)	F	Loin	O8:H19	ehxA/lpf2-2/stx_{2a}
M-E-1-14 (3)	E	Carcass	O8:H7	ehxA/lpf2-2/stx_{2a}
M-D-1-14 (17)	D	Carcass	O91:H21	ehxA/saa/iha/lpf2-2/$stx_{2c(vh-b)}$
M-F-4-14 (6)	F	Heart of rump	ONT:H38	ehxA/saa/iha/lpf2-2/stx_{1a}/stx_{2a}
M-A-4-14 (1)	A	Heart of rump	O46:H38	ehxA/saa/iha/lpf2-2/subAB/stx_{1a}/stx_{2a}
M-B-5-14 (7)	B	Trimmings	O174:H21	iha/lpf2-2/$stx_{2c(vh-b)}$
M-F-4-14 (8)	F	Heart of rump	O174:H21	iha/lpf2-2/$stx_{2c(vh-b)}$
M-G-5-14 (9)	G	Trimmings	O149:H16	ehxA/saa/iha/lpf2-2/subAB/$stx_{2c(vh-b)}$
M-C-1-14 (6)	C	Carcass	O83:H25	iha/lpf2-2/$stx_{2c(vh-a)}$
M-C-1-14 (9)	C	Carcass	ONT:H25	iha/lpf2-2/ subAB/stx2$_{c(vh-a)}$
M-D-1-14 (18)	D	Carcass	ONT:H21	ehxA/saa/iha/lpf2-2/stx_{1a}/stx_{2a}
M-D-2-14 (4)	D	Loin	O15:H27	lpf2-2/stx_{1a}/$stx_{2c(vh-b)}$
M-D-2-14 (7)	D	Loin	O183:H18	ehxA/saa/lpf2-2/subAB/stx_{1a}
M-A-5-14 (2)	A	Trimmings	O171:NM	iha/lpf2-2/stx_{2b}
M-D-3-14 (8)	D	Striploin	O103:H21	eae/ehxA/efa/stx_{1a}/stx_{2a}
M-G-3-14 (10)	G	Striploin	O91:H21	ehxA/saa/lpf2-2/cdt-V/stx_{2a}/$stx_{2c(vh-b)}$
M-G-5-14 (4)	G	Trimmings	O91:H21	ehxA/saa/iha/lpf2-2/stx_{2a}/$stx_{2c(vh-b)}$
M-F-1-14 (1)	F	Carcass	O91:H21	ehxA/saa/iha/lpf2-2/stx_{1a}/stx_{2a}
M-H-1-14 (1)	H	Carcass	O91:H21	ehxA/saa/iha/lpf2-2/stx_{2a}/$stx_{2c(vh-b)}$
